# Chemical Manufactories and Public Hygiene

**Published:** 1858-10

**Authors:** 


Chemical manufactories and public hygiene.

In August 1854, the Belgian Government appointed a commission to investigate the effects of chemical manufactories on the operatives employed, as well as on the vegetation in the neighbourhood. The results of the labours of the commission are given in the *Journal de Chimie*, and in the *American Medical Monthly*, for July 1858. The conclusions of the committee are summed up as follows:

*Influence of Manufactories on Plants.*—"The conclusions of the commission are summed up as follows: 1st. Acid emanations, which escape from manufactories of chemicals, are capable of injuring the growth of certain plants; 2d. Nevertheless, the effects are produced in such an unequal way upon different kinds of ligneous and herbaceous plants, as that certain species appear to resist the harmful influence of the acid gases very well, while others are injured by the same, but to various extents; 3d. Among the last some cease to show any sign of alteration, even at a slight distance from the manufactories, whilst the alteration of others is effected at great distances, but always within certain limits; 4th. The radius of the injurious influence of such acid gases depends on several circumstances essentially different, but which cannot be absolutely determined; although in each given case they can be determined practically by observing at what distance vegetables, which are readily injured by the emanations, cease to present such special alterations as could be ascribed to their action; 5th. The radius of injurious influence, determined in this way, differs very much, not only in different establishments, but even in different directions from the same; and it was always greater in the direction of the dominant winds, whilst in the direction of winds less frequent it was always small and inconsiderable; 6th. In the direction of the dominant winds, the influence did not extend beyond 2,000 metres as a maximum, nor below 600 metres as a minimum.

"*Influence of Manufactories on Population.*—From the data collected by the government for five years, with reference to births and deaths, it appears that the means were favourable to the increase of population, as well in the districts where the manufactories were established as in those adjoining. Thus, from 1839 to 1843 the ratio of deaths was one in sixty-six, and from 1844 to 1848, one in fifty-eight. It is remarkable that no case of cholera had appeared in these manufacturing districts, and that, with the exception of Floreffe, all were exempted from the typhoid epidemics which have prevailed in the province since 1843. During the epidemic of Floreffe, which raged in the hamlet of Buzet, where there were seventy-five sick, only one of the labourers who worked in a chemical manufactory was attacked by the disease, and he had taken a long journey two days before.

"A report of Dr. Cambrelin, president of the Medical Commission of the province of Namur, states, that the emanations from the chemical manufactories of the valley of the Sambre do not give rise to any peculiar affection, and that the diseases of the chest are not now as frequent as formerly in the districts where the manufactories exist. The general health of the neighbouring population remains the same as in the past—even that of the labourers occupied in the manufactories; and if they are ever attacked at the beginning of their apprenticeship with laryngitis, bronchitis, or difficulty in respiration, custom soon causes these indispositions to disappear, without any recurrence. The same observations are made as to the horses employed in the establishments.

"Thus, contrary to expectation, the ratio of mortality is diminished in the midst of these manufactories of chemicals; but should this diminution be attributed to the direct influence which these manufactories have on the health of the workmen and the population? No one would dare to assert this. These manufactories have contributed, in one sense, to this happy result, by extending comfort around them, and offering regular employment, with suitable wages, to a portion of the population. In any point of view, and whatever part we may wish to ascribe to them in the way of direct influence, none of the facts will authorize us to conclude that these manufactories have exercised an *injurious* influence on health, and that they are not a cause of prosperity for the countries in which they have been established."

Of the other books and papers before us, given in the book list, we select a few for brief reference. A book entitled *Health and Disease, their Laws, with Plain Practical Pre-*

*scriptions for the People,* by Benjamin Ridge, M.D., is an elegantly got up work of considerable thickness, and implying a month's hard reading. We are nervously anxious on all occasions to do our duty, without fear or favour, in this department of the Review, and yet instinctively shrink, however urgently it may be called for, from the painful task of writing condemnations or of exciting the feelings of the many against an unfortunate writer, whose only fault may be that of condemning himself in his own book. This feeling, morever, holds the more imperative sway, when it is clear that the mental poverty and not the will of an author supplies the occasion for the critic's display of justice unmoved by mercy. We therefore feel it to be the kindest act, both to ourselves and the book before us, to say the best word for the intention, and to leave the subject matter to reviewers of a sterner mould.

Mr. Peter Hinckes Bird, in a work *On the Nature, Causes, Statistics, and Treatment of Erysipelas,* concludes (*a*) that erysipelas is merely an example on the skin of that diffuse inflammation, which in other tissues constitutes diffuse inflammation of the mucous membrane, diffuse phlebitis, puerperal fever; all of which have a common origin, a poison in the blood, are infectious and contagious, and may mutually produce each other. (*b*). That the term erysipelas should be confined to diffuse inflammation of the skin, and subcutaneous cellular tissue. (*c*). That erysipelas is best treated by stimulants and support; and when complicated with inflammation of the subcutaneous cellular tissue, by early incisions which should extend to the depth of the disease.

Mr. J. C. Clendon, in a reprint *On some Forms of Disease arising from the Retention of Decayed Teeth,* calls attention to the mischiefs which occur in the shape of neuralgia, abscess, contraction of the facial muscles and paralysis of the face, from the presence of teeth in various stages of disease. He declares, that when once a tooth has been the subject of acute inflammation, it is virtually doomed; he is frequently reminded reproachfully, he states, that there are dentists who "never extract teeth; they can stop anything!" and he believes it; for he is often called upon to extract a tooth that has been stopped when in a state of active inflammation; a practice which cannot be in accordance with any principles of science, but must proceed from sheer ignorance, or something worse; for, unfortunately, in dental practice, it is often far more lucrative to suppress the truth than honestly to declare it. Mr. Clendon states, that many complaints which come under the notice of the practitioner as diseases of the body are, in reality, diseases of the teeth, and that there are no parts which more widely and universally affect the general system than the teeth; an observation in which an important truth is exaggerated into positive error.

Four letters to Sir James Clarke, bart, on *Administrative Reform, in Relation to the Medical Schools and the Examining Boards,* although containing a mistake in prophecy, as to the results of Mr. Cowper's Bill, supply much excellent suggestive matter on the subjects of which they treat; we hold the book in store for another and more general purpose.

Reports on the Sanitary Condition of St. Pancras, by Dr. Hillier; on St. Giles, by Dr. Buchanan; on the City of London, by Dr. Letheby; and on Bethnal Green, by Mr. Pearce,—each, medical officer of health for the respective districts,—are instructive documents like all which have appeared from the new order of sanitary officers appointed under Sir Benjamin Hall's Act. We have in progress an analysis of all these documents.

A tract entitled, *Observations on the Process Patented by M. Falcony, for Embalming and Preserving the Deceased,* contains several testimonials, from men of eminent position, bearing on the success of Falcony's application. The chemical preparations used by Mr. Falcony are of two kinds; one is a white powder, mixed with sawdust as a vehicle, and intended only for temporary preservation. The other is a liquid for injecting into the arteries, and is intended for the permanent preservation of the dead body. Mr. Luther Holden, Mr. Stanley, Dr. Ferguson, Mr. Paget, Dr. Baly, and Mr. Tom Taylor, have all borne testimony to the success of these preservations. We have ourselves seen the result of one experiment with the liquid, at the Grosvenor Place School of Medicine, which promises well. We are unable this time to give the exact composition of the antiseptic. It is probably a preparation of zinc.

We must not conclude this notice without congratulating sanitary reformers on the fact, that a new periodical is added to literature in the shape of the *Edinburgh Veterinary Review.* The talented and learned editor of this serial, Mr. John Gamgee, has a full appreciation of the meaning of hygienic medicine, and of the connexions which exist between the diseases of men and animals. Departing from the old routine of bare curative practice, Mr. Gamgee aims at teaching the higher object of veterinary science, namely, the *prevention* of diseases. His first number is admirably arranged, is distinguished by sound literary ability, and if sustained in the same manner will carry with it all the success it deserves.

